# How Important Are the Perceptions and Barriers to Implementing On‐Farm Food Safety Practices Among Salad Vegetable Farmers Evidence From Eastern Region, Ghana

**DOI:** 10.1155/ijfo/2821395

**Published:** 2026-10-08

**Authors:** Regina Ofori Asante, Prosper Kaangmenpuo Kayelle

**Affiliations:** ^1^ Department of Food and Postharvest Technology Koforidua Technical University Koforidua Ghana ktu.edu.gh; ^2^ Department of Business and Social Science Faculty of Agriculture Dalhousie University Truro Nova Scotia Canada dal.ca

**Keywords:** constraints, food safety, Ghana, salad vegetables, vegetable production

## Abstract

This study assessed the perceptions and challenges faced by vegetable farmers in implementing on‐farm food safety practices. Primary data for the analysis were based on survey data from a sample of *N* = 390 farmers in the Eastern Region, a major vegetable production region of Ghana. The data was analyzed using the relative importance index (RII) and problem confrontation index (PCI). Results indicated that farmers recognize and prioritize their responsibility to ensure vegetable safety as the most critical factor (RII = 0.8964; PCI = 1748) in promoting food safety along the salad vegetable production chain. Inadequate resources, such as clean water and equipment, were also identified as significant barriers (RII = 0.8653; PCI = 1683), along with awareness of the risks posed by unclean vegetables produced (RII = 0.8610; PCI = 1679). Although farmers reported strong awareness of safe practices, the PCI results revealed that structural constraints, especially limited access to resources and insufficient training, impede the consistent application of on‐farm food safety measures. Behavioral factors linked to time constraints and motivation to practice hygienic practices were perceived as less problematic but notable. The findings highlight the need for targeted (as opposed to standardized or uniform) interventions combining resource provision, capacity building, and technical education to enhance food safety compliance among vegetable farmers. Addressing the farm‐level gaps is vital to reducing the risk of foodborne illnesses and improving public health outcomes.

## 1. Introduction

The growing consumption of fresh salad vegetables reflects a positive shift toward healthier diets [1]. However, this trend also has associated increased risk of microbial contamination due to the frequent consumption of fresh, ready‐to‐eat produce. Salad vegetables have been repeatedly implicated in foodborne outbreaks globally [2, 3]. The rising reported incidence of foodborne illnesses linked to contaminated produce in Ghana [4], highlights the importance and urgent need to address the root causes of contamination.

Microbial contamination can occur at various stages along the food production and supply chain. Major sources include polluted irrigation water, inadequately composted manure, and poor postharvest handling practices [5]. Studies have reported *Listeria monocytogenes* in 14% of produce samples, *Staphylococcus aureus* in 45.5% of raw vegetables, and *Salmonella* spp. in 6.7% of samples collected from washing and packaging facilities, demonstrating significant public health risks [6]. Pathogens such as *Escherichia coli*, *Salmonella* spp., and *Shigella* spp. are commonly found in vegetables irrigated with contaminated water [7]. Partially composted manure also contributes to microbial loads, and surface washing alone is often ineffective in removing such pathogens [8, 9].

Among Ghanaian smallholder farmers, limited access to resources and training hinders the adoption of good agricultural practices (GAP) [10, 11]. For instance, a study in Accra reported that 70% of farmers used wastewater from drains for irrigation, whereas 99% applied inadequately composted manure practices that compromise food safety and reduce consumer confidence [5]. Although awareness is growing, there remains a lack of empirical research on the specific barriers farmers face in implementing effective food safety measures. Understanding these barriers is essential for designing practical and targeted context‐specific interventions.

Previous studies in Ghana have mainly focused on detecting microbial contaminants in vegetables and irrigation water, emphasizing pathogen identification and public health risks [4, 12]. Some studies have examined hygiene and sanitation awareness, particularly in urban or peri‐urban contexts [13], but few have explored farmers′ perceptions of food safety or identified the practical challenges that hinder safer on‐farm practices, especially among commercial salad vegetable farmers in rural areas.

To address these gaps, this study applied the relative importance index (RII) and problem confrontation index (PCI) to assess barriers to implementing food safety practices among salad vegetable farmers in Ghana′s Eastern Region. The RII quantifies the perceived importance of the barriers considered [14], whereas the PCI measures how severely these issues disrupt daily salad vegetable farming operations [15]. Together, these tools provide a structured framework for evaluating and prioritizing food safety challenges based on both perception and actual food safety impact.

The findings from this empirical study will inform the development of targeted interventions, such as GAP training, safe manure management, and improved irrigation infrastructure. By providing farmers with the technical knowledge and resources needed to reduce contamination risks, the study contributes to efforts to improve the microbial safety of salad vegetables. It contributes to public health and sustainable agriculture in Ghana.

## 2. Materials and Methods

### 2.1. Study Design and Data Collection

Study participants were selected through a combination of purposive and random sampling to ensure diversity of farming systems and representativeness. Agricultural extension officers and community leaders assisted in identifying eligible farmers based on criteria such as farm size and vegetable farm type. Participant recruitment methods included community meetings, local radio announcements, and word‐of‐mouth. Research enumerators conducted farm visits to administer questionnaires directly and assisted respondents with limited literacy.

A total of 400 farmers were approached and interviewed during the study period. After reviewing the completed questionnaires for completeness and consistency, 390 responses were considered valid and included in the final analysis. This study interviewed 400 salad vegetable farmers from 22 districts in the Eastern Region of Ghana. The Eastern Region was selected for several reasons. First, the region is a leading vegetable producing region in the country (along with other smaller locations such as Akomadan area of the Ashanti Region). In addition, the Eastern Region is saddled between the two largest cities in Ghana (i.e., Kumasi and Accra). Accra is the national capital and offers ready market for fresh salad vegetables (commonly sold to restaurants, street food vendors and households). Accra is also home to the largest affluent local residents and expatriate population in Ghana, who constitute the largest segment of salad vegetable consumers in the country. These factors contribute to the dominance of the Region as the leader in vegetable crop production. The participating districts included Kwahu East, Kwahu South, Kwahu West, Fanteakwa North, Fanteakwa South, Birim North, Birim Central, Birim South, Akyemansa, Denkyembour, Kwaebibirem, West Akim, Nsawam Adoagyiri, Ayensuano, East Akim, New Juaben North, New Juaben South, Abuakwa North, Abuakwa South, Yilo Krobo, Lower Manya Krobo, and Asuogyaman (Figure 1). Kwahu Afram Plains North and South districts were not included because they are not traditionally engaged in salad vegetable production.

**Figure 1:**
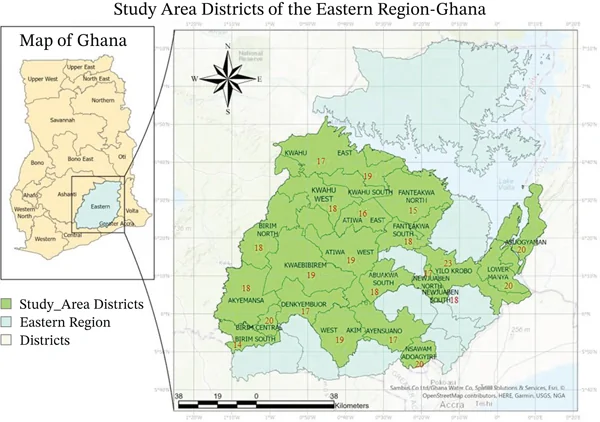
Map showing the study area districts within the Eastern Region of Ghana. Note: The main map highlights selected districts where the study was conducted (shaded in green). Insets include the national map of Ghana with the Eastern Region highlighted, and a regional district map for reference.

The data collection strategy was structured using the knowledge, attitudes, and practices (KAP) framework [16, 17]. A “not sure” response option was included where appropriate to account for possible uncertainty in farm management practices or decisions [18]. The survey questionnaire consisted of four sections. Section 1 had questions to collect demographic data such as age, sex at birth, education, farming experience, and other socioeconomic indicators. Vegetable farm‐related data included crop nutrient sources, irrigation water type, and vegetable types cultivated. Questions in section 2 were used to assess food safety knowledge through various closed‐ended questions with “yes,” “no,” and “not sure” response options. Important food safety knowledge topics included personal hygiene, foodborne illnesses, microorganisms, and sanitation practices. Questions in section 3 elicited behaviors and attitudes using survey items on a five‐point Likert scale ranging from “Always” to “Never,” reflecting farmers′ views on hygiene and safety practices [19]. Section 4 emphasized issues about hygiene and sanitation practices using 13 questions with “yes,” “no,” or “not sure” response options. For scoring purposes, “Yes” responses were assigned a score of 1, whereas “No” responses were assigned a score of 2. Responses of “Not sure” were retained in the original dataset but were excluded from the calculation of the knowledge index because they did not represent a definitive affirmative or negative response and could not be meaningfully assigned to either category without introducing an arbitrary classification. The knowledge index was therefore calculated using the valid “Yes” and “No” responses only. The same scoring procedure was applied consistently across the relevant knowledge items. Ethical approval for the study was obtained from the Humanities and Social Sciences Research and Ethics Committee of Kwame Nkrumah University of Science and Technology (Reference File ID: HuSSREC/AP/102/Vol.3).

Face‐to‐face interviews were conducted using a pilot‐tested questionnaire. The pilot testing was carried out in four randomly selected districts (Ayensuano, East Akim, New Juaben North, and New Juaben South) to validate the instrument prior to full deployment. Trained research assistants administered the final questionnaire between January and April 2024.

### 2.2. Statistical Analysis

The exact population of salad vegetable farmers in the selected districts was not available from a single reliable sampling frame. Therefore, the population was approximated at 10,000 farmers for the purpose of estimating the required sample size using Yamane′s approach at a 5% level of precision [20]. The resulting minimum sample size was approximately 385 respondents. To allow for possible incomplete responses and to enhance representation across the selected districts, 400 farmers were approached and interviewed. Following data screening, 390 questionnaires were complete and valid and were retained for the final analysis.

The survey data was coded and analyzed using IBM SPSS Statistics Version 27.0 [21]. Descriptive statistics, including frequencies and percentages, were used to summarize categorical variables. To assess the reported level of importance assigned by respondents to various food safety factors in salad vegetable production, the RII was calculated for each item, using the equation [14]:

RII=∑inwi A×N,i=12501,,..,, ≤RII≤,

where *w*_*I*_ is the weighted score for each response category, calculated by multiplying the Likert scale value (1–5) by the number of respondents selecting that value (n_i_). A is the highest Likert scale score (5), and *N* is the total number of respondents. The RII ranges from 0 to 1, with higher values indicating greater perceived importance.

Similarly, to evaluate the severity of barriers and vegetable farming challenges encountered, the PCI was calculated using the relationship [15]:

PCI=Pn×5+Pm×4+Pl×3+Pk×2+Pj×1,

where *P*_*n*_ denotes the frequency of responses for “always,” *P*_*m*_ denotes the frequency of responses for “very often,” *P*_*l*_ denotes the frequency of responses for “sometimes,” *P*_*k*_ denotes the frequency of responses for “rarely,” and *P*_*j*_ denotes the frequency of responses for “never.” Higher PCI scores indicate more pressing or heightened barriers or problems faced by farmers. The indices were used to rank factors by their perceived importance and challenge severity. Results were evaluated to identify food safety priority issues associated with vegetable farming for intervention.

To assess the factors, which influence implementation of on‐farm food safety practices, the study applied two complementary tools: the RII, which measures the perceived importance of each factor, and the PCI, which evaluates the severity of barriers faced in practice. Based a five‐point Likert scale, respondents rated each item, and the results were ranked to provide a comprehensive picture of both perceptions and challenges.

## 3. Results and Discussion

### 3.1. Demographic, Farm, and Farming Characteristics of Farmers

Descriptive statistics presented in Table 1 indicate that the majority of participants were male (72%), compared with females (28%). This distribution reflects the broader gender pattern in Ghanaian agriculture [22], suggesting that salad vegetable production follows the general structure of agricultural participation in the country. The predominance of male farmers may have implications for food safety implementation, as gender differences can influence access to agricultural training, resources, and farm‐level decision‐making. Therefore, food safety interventions should incorporate gender‐sensitive approaches to encourage participation of both male and female farmers [23, 24].

**Table 1:** Demographic and farm characteristics, fertilizer usage, vegetable types, and sources of irrigation of participants.

a. **Demographic and farm characteristics of participants**					
**Variable**	**Category**	**No. of participants**		**Percentage (%)**	

Sex	Male	282		72.3	
	Female	108		27.7	

Age group (years)	18–24	8		2.1	
	25–34	49		12.6	
	35–44	107		27.4	
	45–54	126		32.3	
	55–64	71		18.2	
	65 or over	29		7.4	

Education level	Basic education	220		56.4	
	Second cycle education	88		22.6	
	Diploma	12		3.1	
	Bachelor′s degree and above	4		1.0	
	No formal education	66		16.9	

Farm size	1–3 acres of land	8		2.1	
	4–5acres of land	156		40.0	
	6–10 acres of land	107		27.4	
	More than 10 acres of land	119		30.5	

Number of workers employed	None	57		14.6	
	1–5 workers	228		58.5	
	6–10 workers	80		20.5	
	More than 10	25		6.4	

Years of farming experience	Less than 1 year	5		1.3	
	1–5 years	57		14.6	
	More than 5 years	328		84.1	

b. **Type of fertilizer, vegetables and sources of irrigation**					

Type of fertilizer used	Organic manure	131		33.6	
	Synthetic fertilizer	209		53.6	
	Both	50		12.8	

Types of vegetables grown	Salad vegetables	100		25.6	
	Both (salad vegetables and other vegetables)	290		74.4	

		**Number of participants**			
		**Yes**	**%**	**No**	**%**

Specific types of vegetables cultivated	Cabbage	273	70.0	117	30.0
	Lettuce	74	19.0	316	81.0
	Green pepper	121	31.0	269	69.0
	Carrots	47	21.1	343	87.9
	Cucumber	78	20.0	312	80.0
	Tomatoes	269	69.0	121	31.0
	Other vegetables	293	75.1	97	24.9

Source of irrigation	Rainwater	293	75.1	97	24.9
	Small reservoirs	43	11.0	347	89.0
	Dams	62	15.9	328	84.1

The largest proportion of farmers were aged 45–54 years (32%), followed by those aged 35–44 years (27%) and 55–64 years (18%). Younger farmers aged 18–24 years represented only 2% of respondents, which is consistent with declining youth engagement in agriculture in Ghana [25]. More than half of the farmers had basic education (56%), whereas 23% had secondary education and 17% had no formal education. The low proportion of farmers with tertiary education suggests that limited formal education may constrain adoption of advanced production technologies and food safety practices. Practical extension training and demonstration‐based learning may therefore be important strategies for improving compliance with GAP [26, 27].

Farm size distribution showed that 40% of farmers cultivated 1.6–2.0 ha, whereas 27% managed 2.4–4.0 ha. About 31% cultivated more than 4.0 ha, whereas 2% cultivated 0.4–1.2 ha. The majority employed between 1–5 workers (59%), indicating that salad vegetable production is largely characterized by small‐ and medium‐scale farming operations. Most farmers (84%) had more than 5 years of production experience, suggesting that vegetable production in the study area is dominated by experienced farmers with considerable local knowledge. However, experienced farmers may require targeted incentives and training to encourage adoption of improved food safety practices [28, 29]. The limited participation of new entrants may reflect barriers related to land access, production costs, and limited youth involvement in agriculture [30].

Most farmers (74%) cultivated a combination of salad and other vegetables, whereas 26% specialized in salad vegetables. Crop diversification is commonly practiced by Ghanaian farmers as a strategy for income diversification and livelihood sustainability [31]. Cabbage (70%) and tomatoes (69%) were the most commonly cultivated vegetables, whereas lettuce (19%), carrot (12%), cucumber (20%), and green pepper (31%) were less frequently grown. Other vegetables cultivated included garden eggs, okro, pepper, and pumpkin.

Regarding farm inputs, 54% of farmers used inorganic fertilizers, 34% used organic manure, and 13% used both. The use of untreated organic manure may increase the risk of microbial contamination because animal waste can harbor pathogens such as *E. coli* and *Salmonella* spp. [4, 32]. Rainwater was the main irrigation source (75%), followed by streams and dams (16%) and reservoirs/dugouts (11%). Although rainfed production remains common in Ghanaian agriculture [33], dependence on rainfall exposes vegetable production systems to climate‐related risks [34].

### 3.2. Perceptions and Barriers to Implementing On‐Farm Food Safety Practices

The findings indicate that farmers generally demonstrated strong awareness and positive perceptions regarding food safety (Table 2). The statement “Farmers are responsible for the safety of vegetables produced” received the highest RII (0.8964) and PCI (1748), indicating recognition among farmers that their production practices influence the safety of fresh vegetables.

**Table 2:** Relative importance index (RII) assessment of perceived barriers to food safety practices in salad vegetables.

Item	*W*_*1*_^a^	*W*_*2*_	*W*_*3*_	*W*_*4*_	*W*_*5*_	Total (*N*)	RII	Rank
Farmers are responsible for the safety of vegetables	2	7	50	73	258	390	0.8964	1
Do you think inadequate resources such as water and equipment hinder the implementation of food safety practices	3	4	67	104	211	389	0.8653	2
Unclean or soiled vegetables can cause food poisoning	5	3	73	96	213	390	0.8610	3
On‐farm practices impact vegetable safety	1	2	83	103	201	390	0.8569	4
Do you think that lack of training in hygienic handling may hinder the ability to observe food safety practices	8	8	91	81	202	390	0.8364	5
Inadequate education regarding the hygienic handling of vegetables hinders the implementation of food safety measures	8	6	101	92	183	390	0.8236	6
Does time constraint obstruct the adherence to hygienic practices	18	10	88	66	205	387	0.8222	7
Does a lack of motivation act as a barrier to practicing proper food hygiene	16	7	86	98	181	388	0.8170	8

^a^ *W_i_* denotes frequency of Likert scale responses where *W_1_* = *never*, *W_2_* = *rarely* = 2, *W_3_* = *sometimes*, *W_4_* = *very often*, *W_5_* = *always*.

### 3.3. RII Analysis

The RII values for all assessed factors were high, ranging from 0.8170 to 0.8964, indicating strong agreement among respondents. Farmers′ responsibility for vegetable safety ranked highest (RII = 0.8964), followed by inadequate resources such as water and equipment (RII = 0.8653). Other highly rated factors included awareness that unclean vegetables may cause food poisoning (RII = 0.8610) and recognition that on‐farm practices influence vegetable safety (RII = 0.8569).

Although training‐related factors, including lack of food safety training (RII = 0.8364) and inadequate education (RII = 0.8236), ranked lower, their relatively high scores indicate that knowledge and skills remain important constraints. Time constraints (RII = 0.8222) and lack of motivation (RII = 0.8170) were ranked lowest but remained relevant barriers.

The high ranking of farmer responsibility suggests strong awareness of the role farmers play in preventing contamination. Similar findings were reported by Amponsah et al. [35], who found that participation in field trials improved Ghanaian vegetable farmers′ awareness of approaches for reducing microbial contamination. However, awareness alone may not guarantee compliance unless farmers have access to adequate resources, incentives, and institutional support.

Limited access to irrigation water, machinery, and farm equipment was identified as an important challenge. Gbedemah et al. [36] reported contamination risks among vegetables produced using unsafe irrigation sources in Accra, highlighting the importance of improved access to safe water and farm infrastructure. Thus, resource limitations may prevent farmers from translating food safety knowledge into practice [5, 10].

Farmers′ recognition that unclean vegetables can cause food poisoning indicates awareness of food safety risks. This observation is consistent with findings from recent research in North Africa, where a gap between food safety knowledge and actual practices was also reported among food handlers, indicating that awareness alone may not always translate into appropriate food safety behaviors [37]. However, previous studies have shown that farmers may lack detailed knowledge of contamination pathways, particularly regarding irrigation water and manure use [38]. Similarly, inadequate training and hygiene knowledge have been linked to food safety challenges in Ghana [39]. This highlights the need for practical training programs that strengthen both knowledge and implementation of safe practices.

Although training and education barriers ranked moderately high, they remain important areas for intervention. Sarfo et al. [40] similarly identified limited food safety training among food handlers in Ghana and recommended regular capacity‐building programs. Therefore, farmer‐focused training and extension services should form an important component of food safety improvement strategies.

The RII rankings provide descriptive information on farmers′ perceptions. Although higher RII values indicate greater perceived importance, differences between ranks should not be interpreted as statistically significant because no inferential statistical test was conducted.

### 3.4. PCI Analysis

The PCI values ranged from 1585 to 1748 (Table 3), indicating varying levels of perceived challenges affecting food safety implementation. The highest PCI was associated with farmers′ responsibility for vegetable safety (1748), followed by inadequate resources such as water and equipment (1683). These findings suggest that farmers recognize their role in ensuring safe production but face practical limitations that restrict implementation.

**Table 3:** Problem confrontation index (PCI) assessment of perceived barriers to food safety practices in salad vegetables.

Item	*P*_*j*_^*a*^	*P*_*k*_	*P*_*l*_	*P*_*m*_	*P*_*n*_	Total (*N*)	PCI	Rank
Farmers are responsible for the safety of vegetables	2	7	50	73	258	390	1748	1
Do you think inadequate resources such as water and equipment hinder the implementation of food safety practices	3	4	67	104	211	389	1683	2
Unclean or soiled vegetables can cause food poisoning	5	3	73	96	213	390	1679	3
On‐farm practices impact vegetable safety	1	2	83	103	201	390	1671	4
Do you think that lack of training in hygienic handling may hinder the ability to observe food safety practices	8	8	91	81	202	390	1631	5
Inadequate education regarding the hygienic handling of vegetables hinders the implementation of food safety measures	8	6	101	92	183	390	1606	6
Does time constraint obstruct the adherence to hygienic practices	18	10	88	66	205	387	1591	7
Does a lack of motivation act as a barrier to practicing proper food hygiene	16	7	86	98	181	388	1585	8

^a^ *P_i_* denotes frequency of Likert scale responses where *P*_j_ = *never*, *P_k_* = *rarely*, *P_l_* = *sometimes*, *P_m_* = *very often*, *P_n_* = *always*.

Previous studies have shown that improved awareness among Ghanaian vegetable farmers can enhance compliance with food safety practices [41]. However, responsibility without adequate resources may create challenges for farmers attempting to adopt recommended practices. Similarly, contamination risks associated with unsafe water sources and inadequate farm infrastructure reported by Gbedemah et al. [36] support the importance of improving access to farm resources.

Recognition that unclean vegetables may cause food poisoning (PCI = 1679) and that on‐farm practices affect vegetable safety (PCI = 1671) demonstrates farmers′ awareness of food safety risks. However, knowledge gaps may still limit consistent adoption of safe practices. This agrees with findings from Tuglo et al. [42], who identified inadequate food safety knowledge and training as major contributors to poor food handling practices in Ghana.

Training (PCI = 1631) and education (PCI = 1606) were identified as important constraints, supporting previous findings that regular food safety education is required to improve hygiene practices among food system actors [43]. Lower PCI values for time constraints (1591) and lack of motivation (1585) indicate that these were perceived as relatively less severe barriers. However, closely ranked PCI values should be interpreted cautiously because they represent relative priority rather than statistically significant differences.

Economic factors may also influence farmers′ willingness to invest in food safety. Mensah et al. [44] reported that improved farm profitability was associated with greater compliance with safety measures. Therefore, interventions combining training, improved access to resources, and economic incentives may enhance adoption of safe vegetable production practices [44, 45].

The RII and PCI produced broadly similar rankings across the assessed food safety practices. Although this similarity may suggest overlapping conclusions, the two indices provide complementary information. The RII reflects the relative importance or perceived significance that respondents attach to the assessed food safety practices, whereas the PCI provides an indication of the level of reported compliance with those practices. Thus, the RII provides insight into how farmers perceive the importance of food safety practices, whereas the PCI provides insight into the extent to which these practices are reportedly implemented. The similarity in rankings suggests that the practices perceived as more important were generally also reported at relatively higher levels of compliance. Using both indices therefore provides a more comprehensive interpretation than relying on either measure alone.

## 4. Conclusions

The RII and PCI analyses provided insights into farmers′ perceptions and the barriers affecting safe vegetable production. Farmers demonstrated a strong sense of responsibility toward food safety; however, implementation of safe practices was constrained mainly by limited access to resources, inadequate training, and insufficient technical support. These findings highlight the need for targeted interventions to improve farmers′ capacity and access to essential resources for strengthening food safety practices in salad vegetable production. Although time constraints and lack of motivation were ranked lower among the identified barriers, they may still influence farmers′ ability to implement food safety practices consistently. For example, some farmers may perceive activities such as regularly cleaning farm equipment, maintaining production records, or treating irrigation water as additional tasks that require extra time beyond routine farming. Similarly, farmers who do not perceive immediate economic benefits from adopting food safety measures may have limited motivation to invest additional effort and resources in these practices. Together, the RII and PCI analyses reveal a critical gap between knowledge and actual implementation. Although farmers understand the importance of food safety and their role in reducing contamination, many lack the resources, technical knowledge, and institutional support necessary for compliance.

## 5. Recommendations

To address challenges related to irrigation water availability and quality, interventions such as improving access to reliable water sources, including options such as solar‐powered mechanized boreholes where feasible, could be considered. This recommendation is proposed as a potential solution to support safe vegetable production and was not directly measured as a farmer preference in this study. The Food and Drugs Authority and other relevant stakeholders should strengthen regular training on GAP, food hygiene, and contamination prevention through collaboration with NGOs, extension officers, and agrifood agencies. Improving farmers′ access to subsidized inputs and sanitation facilities, such as protective clothing and on‐farm wash stations, is essential for effective implementation. Incentive‐based approaches, including food safety certification linked to improved market opportunities, may encourage compliance. Community awareness campaigns and stronger alignment with national food safety policies will further support sustainable improvements in food safety practices.

## 6. Limitation

A limitation of this study is the absence of subgroup analysis to examine differences in food safety perceptions and constraints across farmer characteristics. Consequently, it was not possible to determine whether these perceptions and constraints varied according to factors such as gender, education, farming experience, or farm size. Future research should investigate these subgroup differences to provide more targeted evidence for the development of interventions and policies aimed at improving food safety practices among salad vegetable farmers.

## Author Contributions

**Regina Ofori Asante:** conceptualization, investigation, data curation, project administration, writing original draft. **Prosper Kaangmenpuo Kayelle:** methodology, formal analysis, writing – review and editing.

## Funding

No funding was received for this manuscript.

## Conflicts of Interest

The authors declare no conflicts of interest.

## Data Availability

The data that support the findings of this study are available from the corresponding author upon reasonable request.
